# Critical analysis of treatment practice of children with conduct disorders at the Belarusian mental health centre

**DOI:** 10.1192/j.eurpsy.2026.11665

**Published:** 2026-07-31

**Authors:** U. Pikirenia, Y. Lapchuk

**Affiliations:** 1 Alcohol and substance use department, Psychiatric Hospital in Frombork, Frombork, Poland; 2 Child department, Minsk Regional Clinic Centre “Psychiatry-narcology”, Minsk, Belarus

## Abstract

**Introduction:**

Conduct disorders with emotional symptoms (ICD-10 F92.8) are prevalent causes of child and adolescent psychiatric admissions. International guidelines prioritise psychosocial interventions and caution against routine pharmacotherapy. We assessed real-world practices in a national referral centre against national and international recommendations.

**Objectives:**

To evaluate inpatient treatments and discharge recommendations for children with F92.8 and quantify concordance with national (Belarus) and international guidance.

**Methods:**

Retrospective audit of discharge summaries (2019–2023) received from Belarusian National Mental Health Centre of 30 pediatric patients (aged 9–15, 63% female) diagnosed with F92.8 was conducted. Extracted variables: age, sex, length of stay, comorbidity, receipt of psychotherapy, and prescribed medications. Descriptive statistics were calculated. The audit complied with local regulations for analysis of anonymised clinical records.

**Results:**

Thirty patients (63% female), median age 14 years (IQR 9–15), median stay 26 days (IQR 22–35); 63% had recorded comorbidities.

Psychotherapy during admission was documented in 40%; outpatient psychotherapy was recommended in 66.7%.

All patients received medication; the median number of concurrent drugs was 3 (IQR 2–3.75).

Antipsychotics were prescribed in 73.3% of cases, including agents not approved for paediatric use or outside local protocol (e.g., quetiapine 27.3%, olanzapine 4.5%); protocol-listed agents (periciazine 45.5%, chlorprothixene 13.6%) predominated, clozapine was not used.

Antidepressants were prescribed in 33.3%; 70% of these prescriptions (notably fluvoxamine and paroxetine) fell outside national recommendations/labels for this age group.

Antiepileptic drugs were given to 53.3% (oxcarbazepine and valproate), frequently without a relevant comorbidity.

Nootropics and vitamins were prescribed in 53.3% (phenibut 56.3% of nootropic recipients), despite limited evidence of benefit and potential safety concerns. Overall, practice showed high polypharmacy and a shortfall in documented psychosocial care relative to guidance.

**Conclusions:**

Treatment strategies at the Centre exhibited significant deviations from national and international guidelines, notably an excessive reliance on polypharmacy and inadequate psychotherapy implementation.

Service realignment is warranted: routine provision and documentation of family-centred psychosocial interventions should be prioritised; pharmacotherapy, when indicated, should follow age-appropriate labels and guidelines, favour monotherapy, and target comorbidity.

These data may inform quality-improvement initiatives and guideline implementation efforts.

**Disclosure of Interest:**

None Declared

